# Prior acute ozone injury dampens Th2 responses to subsequent repetitive ozone exposures in mice

**DOI:** 10.21203/rs.3.rs-8289926/v1

**Published:** 2025-12-09

**Authors:** Kshitz Paudel, Sonika Patial, Yogesh Saini

**Affiliations:** Department of Population Health and Pathobiology, College of Veterinary Medicine, North Carolina State University; Comparative and Molecular Pathogenesis Branch, Division of Translational Toxicology, National Institute of Environmental Health Services; Department of Population Health and Pathobiology, College of Veterinary Medicine, North Carolina State University

**Keywords:** Ozone, Lung, Mice, Acute Lung Injury, Th2 inflammation

## Abstract

Ground-level ozone (O_3_), a criteria air pollutant, can cause significant adverse effects on lung health, including airway inflammation, compromised lung function, and increased susceptibility to lung infections. This study was conceived to determine whether a history of high-concentration O_3_-induced acute lung injury responsiveness of respiratory tract to low-concentration repetitive O_3_ exposures. Accordingly, we hypothesized that prior acute O_3_ exposure would modulate the lung’s Th2 responses to subsequent repetitive O_3_ exposures. We exposed 8–10-week-old C57BL6/J mice to either filtered air (FA) or 2 ppm O_3_ for three hours, followed by a three-week recovery period, after which the mice received daily exposures to FA or 1 ppm O_3_ for four hours over 9 days. We evaluated immune cell recruitment, inflammatory mediators in cell-free bronchoalveolar lavage fluid (BALF) and examined mucous cell metaplasia and epithelial cell injury in lung tissue sections. As expected, FA-FA (no O_3_ exposure) mice did not exhibit any signs of injury or inflammation. The O_3_-FA (acute O_3_ exposure only) groups exhibited baseline immune cell populations and no evidence of MCM suggesting almost complete recovery from acute lung injury. In contrast, FA-O_3_ group (repetitive O_3_ exposure only) demonstrated loss of body weight, marked immune cell recruitment, and prominent MCM. These mice also displayed elevated BALF levels of eotaxin, IL-1α, IL-1β, and IL-4, along with increased number of mast cells and FIZZ1^+^ epithelial cells in the lungs. Compared with FA-O_3_ group, the O_3_-O_3_ group (both acute and repetitive O_3_) exhibited attenuated responses, as evidenced by diminished eosinophil and lymphocyte counts, and attenuated MCM. BALF analysis revealed lower levels of eotaxin, IL-1α, IL-1β, and IL-4, but elevated levels of G-CSF, KC, IL-6, IL-10, and IL-12 in the O_3_-O_3_ group. Furthermore, these mice displayed reduced numbers of mast cells and FIZZ1^+^ epithelial cells. These findings suggest that prior exposure to acute high-concentration O_3_ modulates inflammatory and remodeling responses induced by subsequent repeated low-concentration O_3_ exposures. The findings from this study highlight the health impacts of O_3_ pollution, particularly in populations experiencing intermittent high-level exposures.

## Introduction

Ground-level ozone (O_3_), a secondary air pollutant, produced as a result of a photochemical reaction of various automobile and industrial byproducts, such as oxides of nitrogen (NOx) and volatile organic compounds (VOCs), in the presence of sunlight. This phenomenon peaks during hot, sunny days, especially in urban areas with high traffic (1). Ground-level O_3_ is a major public-health problem, with ~ 490,000 deaths attributed to O_3_ exposure worldwide in 2021 (2). Ground-level O_3_ irritates the respiratory system triggering airway injury and inflammation, which can result in structural airway remodeling and reduced lung function (3, 4). Exposure to O_3_ also exacerbates preexisting lung conditions, such as asthma and chronic obstructive pulmonary diseases (COPD), increasing severity of symptoms and reducing responsiveness to conventional therapies (5, 6). Despite these health concerns, our understanding of the respiratory disturbances caused due to O_3_ is limited.

Acute versus repetitive O_3_ exposures produce different pathological outcomes in both humans and mice. Further, different O_3_ concentrations in acute or repetitive paradigm may also result in different severity of disease outcomes. Acute O_3_ exposure in humans and mice generally triggers a rapid neutrophilic inflammatory response together with airway epithelial cell injury in humans and mice (7–10). TLR4-dependent signaling has been implicated in mediating aspects of the acute O_3_-induced neutrophilic response (11). Acute O_3_ exposure also results in the generation of reactive oxygen species (ROS), which induces oxidative stress, and disrupts the respiratory epithelial barrier in mice (12–14). In contrast, repetitive O_3_ exposure in mice triggers type-2 (Th2)-like inflammation which is characterized by eosinophilic inflammation, mucous cell metaplasia (MCM), and extracellular matrix remodeling (15, 16).

Because ground-level O_3_ concentration and exposure duration can fluctuate unpredictably, the respiratory response to a given O_3_ exposure may be influenced by prior exposure. Previous acute lung injury can alter responses to later insults (17, 18). For example, lipopolysaccharide (LPS)-induced acute lung injury has been shown to dampen a subsequent house dust mite (HDM)-induced mucoinflammatory phenotype in mice (18). Similarly, repetitive O_3_ exposure (four doses) have been reported to attenuate the severity of lung injury caused by a subsequent acute O_3_ exposure in mice (19). However, whether an initial acute O_3_-induced lung injury modifies the development of a subsequent, repetitive O_3_-induced mucoinflammatory phenotype has not yet been investigated. To compare the lung’s response to repetitive O_3_ exposure in mice with or without previous acute O_3_ exposure, we hypothesized that prior acute O_3_ exposure would modulate the lung’s Th2 responses to subsequent repetitive O_3_ exposures. Accordingly, we exposed 8–10-week-old C57BL6/J mice to either 2ppm O_3_ or filtered air (FA) for 3 hours. After a 3-week recovery period, mice were repetitively exposed to 1ppm O_3_ or FA for nine days (4h/day). We evaluated the recruitment of immune cells into the airspaces, markers of lung epithelial injury, concentrations of inflammatory mediators in cell free bronchoalveolar lavage fluid (BALF), and MCM responses. The findings from this study offer new insights into how an initial high-concentration O_3_-induced acute lung injury and inflammation alters airway responses induced by subsequent repetitive low-concentration O_3_ exposures, with implications for understanding the susceptibility to lung diseases induced by repetitive O_3_-exposures.

## Methods

### Animal Husbandry, Experimental design, and ozone (O_3_) exposure

Eight-week-old C57BL/6J female mice were procured from the Jackson Laboratory (Bar Harbor, ME) and were acclimatized for 2 weeks in the Division of Laboratory Animal Medicine vivarium at Louisiana State University (LSU). Mice were maintained in individually ventilated, hot-washed polycarbonate cages on a 12-hour light-dark cycle and provided with a regular diet and water *ad libitum*. All animal use protocols were approved by the LSU Institutional Animal Care and Use Committee (IACUC).

**Figure 1.**
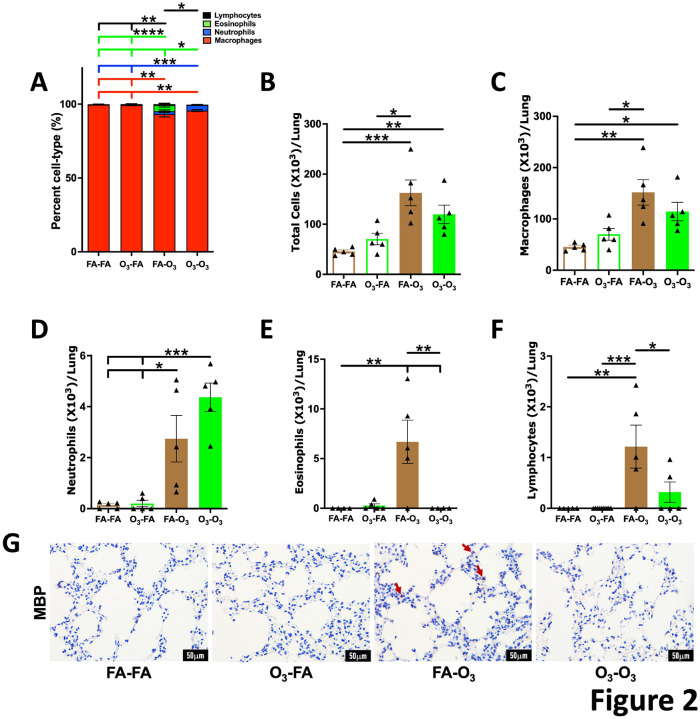
**Figure 1. Prior acute O_3_ exposure attenuates subsequent repetitive O_3_-induced changes in body weight: (A)** Experimental design depicting the O_3_ exposure regimen across different experimental cohorts. **(B)** Body weight gain during 21 days of recovery period following exposure to FA (solid blue line, *n* = 10) or acute O_3_ (solid red line, *n* = 10). **(C)** Body weight gain during 9 doses of FA or repetitive O_3_ in mice previously exposed to FA (FA-FA; dotted blue line, *n*= 5 and FA-O_3_; solid blue line, *n* = 5). Body weight gain during 9 doses of FA or repetitive O_3_ in mice previously exposed to acute O_3_ (O_3_-FA; dotted red line, *n* = 5 and O_3_-O_3;_ solid red line, n=5). Significant differences between FA-FA vs FA-O_3_ (blue asterisks), O_3_-FA vs O_3_-O_3_ (red asterisk), and FA-O_3_ vs O_3_-O_3_ (black asterisks) are shown. *p<0.05; **p<0.01; ****p<0.0001; using one way ANOVA followed by Tukey’s multiple comparison post hoc test.

**Figure 2.**
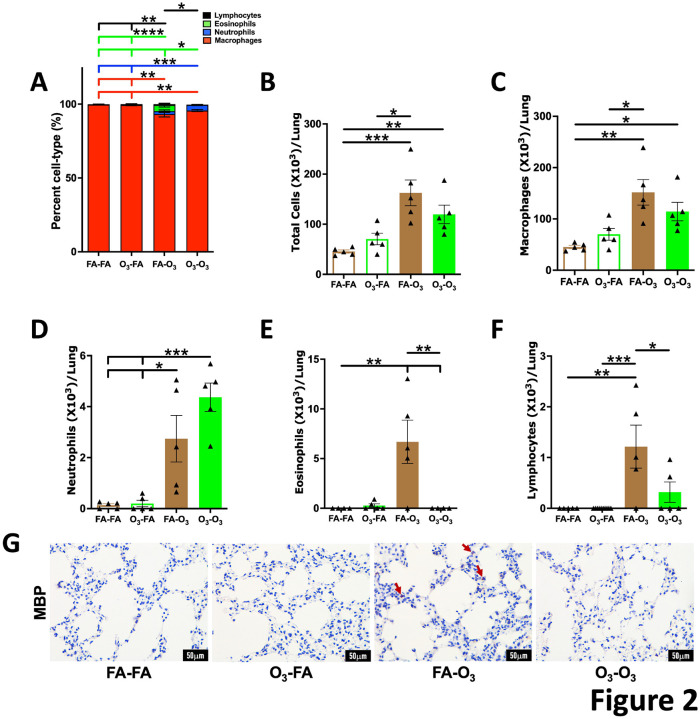
**Figure 1. Prior acute O_3_ exposure attenuates subsequent repetitive O_3_-induced changes in body weight: (A)** Experimental design depicting the O_3_ exposure regimen across different experimental cohorts. **(B)** Body weight gain during 21 days of recovery period following exposure to FA (solid blue line, *n* = 10) or acute O_3_ (solid red line, *n* = 10). **(C)** Body weight gain during 9 doses of FA or repetitive O_3_ in mice previously exposed to FA (FA-FA; dotted blue line, *n*= 5 and FA-O_3_; solid blue line, *n* = 5). Body weight gain during 9 doses of FA or repetitive O_3_ in mice previously exposed to acute O_3_ (O_3_-FA; dotted red line, *n* = 5 and O_3_-O_3;_ solid red line, n=5). Significant differences between FA-FA vs FA-O_3_ (blue asterisks), O_3_-FA vs O_3_-O_3_ (red asterisk), and FA-O_3_ vs O_3_-O_3_ (black asterisks) are shown. *p<0.05; **p<0.01; ****p<0.0001; using one way ANOVA followed by Tukey’s multiple comparison post hoc test.

**Figure 3.**
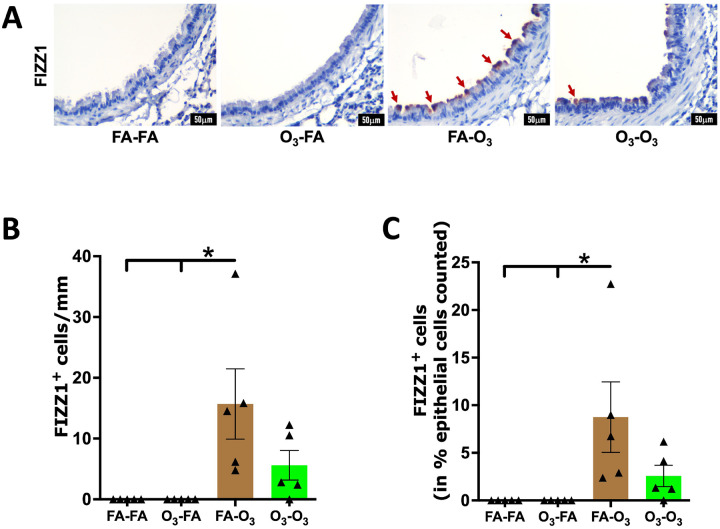
**Prior acute O_3_ exposure mitigates the increase in FIZZ1 expression in repetitively O_3_-exposed mice: (A)**Representative photomicrographs showing the immunolocalization of FIZZ1 in left lung lobe sections from mice in FA-FA, O_3_-FA, FA-O_3_, and O_3_-O_3_ experimental groups. Quantification of FIZZ1-positive cells per millimeter of basement membrane **(B)** and percent FIZZ1-stained cells **(C)** from mice in FA-FA (open brown bar, *n* = 5), O_3_-FA (open green bar, *n* = 5), FA-O_3_ (solid brown bar, *n* = 5), and O_3_-O_3_ (solid green bar, *n* = 5) experimental groups. Error bars represent SEM. *p<0.05 using one-way ANOVA followed by Tukey’s multiple comparison post hoc test. FIZZ1, found in inflammatory zone 1. Scale: 50mm. Magnification: 400X.

**Figure 4.**
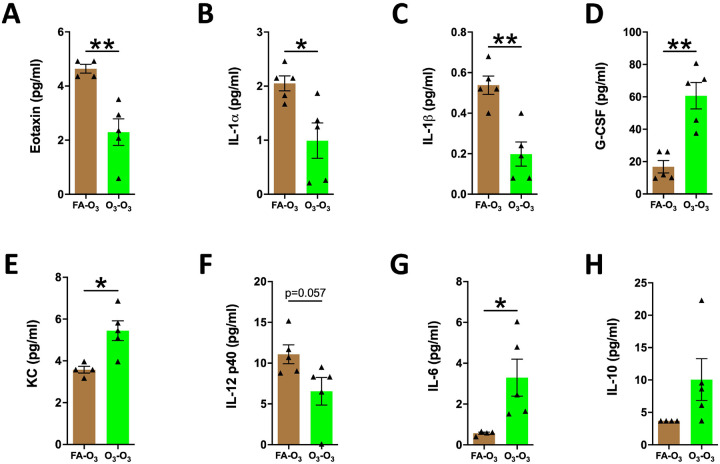
**Prior acute O_3_ exposure alters the levels of inflammatory mediators in airspaces of repetitively O_3_-exposed mice:** Cytokine levels (picogram per milliliter) of eotaxin **(A)**, IL-1a **(B)**, IL-1b **(C)**, G-CSF **(D)**, KC (CXCL1) **(E)**, IL-12 p40 **(F)**, IL-6 **(G)**, IL-10 **(H)** in mice in FA-O_3_ (solid brown bar, *n* = 4–5) and O_3_-O_3_ (solid green bar, *n* = 4–5) experimental groups. Error bars represent SEM. *p<0.05; **p<0.01; using Student’s t test. IL-1a, interleukin-1 alpha; IL-1b, interleukin-1 beta; G-CSF, granulocyte-colony stimulating factor; KC, keratinocyte chemoattractant; IL-12 p40, interleukin-12 subunit p40; IL-6, interleukin-6; IL-10, interleukin-10.

Mice were randomly assigned to four experimental groups: 1) Group 1, FA-FA: Mice were exposed to filtered air (FA) for 3 hours, followed by a 3-week recovery period, and were then exposed repetitively to FA (9 days; 4 hours/day) (Fig. 1A); 2) Group 2, O_3_-FA: Mice were exposed to O_3_ (2ppm) for 3 hours, followed by a 3 week recovery period, and were then exposed repetitively to FA (9 days; 4 hours/day) (Fig. 1A); 3) Group 3, FA-O_3_: Mice were exposed to FA for 3 hours, followed by a 3 week recovery period, and were then exposed repetitively to O_3_ (9 days; 1ppm at 4 hours/day) (Fig. 1A); 4) Group 4, O_3_-O_3_: Mice were exposed to O_3_ (2ppm) for 3 hours, followed by a 3 week recovery period, and were then exposed repetitively to O_3_ (9 days; 1ppm at 4 hours/day) (Fig. 1A).

O_3_ generation and data acquisition were done as previously described (16). Briefly, O_3_ was generated using an O_3_ generator (TSE Systems, Chesterfield, MO) and its concentration was continuously monitored in real-time with a UV photometric O_3_ analyzer (Envia Altech Environment, Geneva, IL). Data were acquired through DACO monitoring and control software (TSE Systems, Chesterfield, MO). Mice that were designated for FA exposures were exposed to filtered air (FA) in identical chambers. Since mice are nocturnal and exhibit higher activity during the night (20), O_3_ and FA exposures were conducted at night to simulate real-life scenarios of increased human activity phases.

### Bronchoalveolar lavage and sample harvesting

Bronchoalveolar lavage (BAL) and sample harvesting were conducted as previously described (16). Within 12–16 hours after the last exposure, mice were anesthetized with an intraperitoneal injection of 2,2,2-tribromoethanol (250 mg/kg; Sigma-Aldrich, St. Louis, MO). After exsanguination, a 20G stub adapter was inserted into the trachea and secured with a suture. The left main stem bronchus was ligated with a suture to prevent lavaging. The right lung was lavaged with a calculated volume [(body weight in grams/2 × 0.035 × 1000 = volume in μL] of ice-cold Dulbecco’s phosphate-buffered saline (DPBS) without calcium and magnesium. BAL fluid (BALF) was centrifuged at 500 × *g* for 5 minutes at 4°C. Cell-free supernatants were stored at −80°C for protein and cytokine analysis. Cell pellets were resuspended in 500 μL DPBS for total cell counts and differential staining. The unlavaged left lung lobe was stored in 10% neutral buffered formalin for histopathological analyses and immunohistochemistry, while the right lung lobes were snap-frozen and stored at −80°C.

### Histopathological Analysis

Formalin-fixed, unlavaged left lung lobes, were paraffin-embedded, sectioned at a thickness of 5μm, and processed for histopathological examination. Hematoxylin and Eosin (H&E)-stained slides were used to assess histopathological changes, while Alcian blue-periodic acid Schiff (AB-PAS) staining was employed to evaluate mucopolysaccharide contents in epithelial cells and airway lumens. The slides were observed with ECLIPSE Ci-L microscope, and photomicrographs were captured via a DS-Fi2 camera attachment (Nikon, Melville, NY).

### Immunohistochemistry

The immunohistochemical localization of major basic protein (MBP), mucin 5B (MUC5B), and found in inflammatory zone 1 (FIZZ1) was performed using previously published methods (21, 22). Briefly, lung sections were deparaffinized with Citrisolv (Decon Labs Inc., King of Prussia, PA), rehydrated through graded series of ethanol solutions (100%, 95%, 70%, 30%), and then processed for antigen retrieval. For MBP, antigen retrieval was performed by incubating the sections with Proteinase K at 37°C for 20 minutes. For MUC5B and FIZZ1, a citrate buffer-based heat-induced antigen retrieval method was used, where slides were heated in 10 mM tri-sodium citrate dihydrate solution with 0.05% Tween 20 (pH 6.0) at 95–100°C for 30 minutes. Endogenous peroxidases were quenched with 3% hydrogen peroxide for 10 minutes at room temperature. Sections were then blocked with a blocking buffer for 20 minutes and incubated with primary antibodies: rat monoclonal MBP (MT-14.7.3; Mayo Clinic, Scottsdale, AZ), rabbit monoclonal FIZZ1 (ab39626; ABCAM Cambridge, MA), and rabbit polyclonal MUC5B (UNC223, University of North Carolina, Chapel Hill, NC) for 1 hour at room temperature. This was followed by two washes through wash buffer. The sections were then rinsed in PBS and processed using VECTASTAIN Elite ABC HRP Kits (PK-6101 (Rabbit) and PK-6104 (Rat); Vector Laboratories, Burlingame, CA) and chromogenic substrate conversion with ImmPACT NovaRED HRP Substrate Kit (SK-4800; Vector Laboratories, Burlingame, CA). Sections were rinsed in tap water and counterstained with Gill’s Hematoxylin-I (EMD Millipore Corporation, Burlington, MA), dehydrated through graded ethanol series, and mounted with VectaMount mounting media (H-5000; Vector Laboratories, Burlingame, CA). Imaging was done using an ECLIPSE Ci-L microscope, and photomicrographs were captured with a DS-Fi2 camera attachment (Nikon, Melville, NY).

### Cytokine analyses

Cell-free BALF was assayed for mouse eotaxin, G-CSF, KC, IL-1α, IL-1β, G-CSF, KC/CXCL1, IL-12p40, IL-6, IL-10, and IL-4 using a Luminex XMAP-based assay, following the manufacturer’s protocol (Bio-Rad, Hercules, CA).

### Statistical Analysis

One-way Analysis of Variance (ANOVA) followed by Tukey’s post hoc test for multiple comparisons was used to determine significant differences among multiple groups. Grubbs’ test was used to identify and remove outliers. The data were expressed as mean ± SEM. Significant differences between two groups were determined with Student’s t-test. The p value less than or equal to 0.05 was considered statistically significant. Statistical analyses were performed using GraphPad Prism 10.2.3 (GraphPad Software, La Jolla, CA).

## Results

### Prior exposure to acute ozone attenuates repetitive O_3_-induced changes in body weight.

To investigate the effects of previous acute O_3_ exposure on lung’s response to repetitive O_3_ exposure, eight-week-old female mice were assigned to four experimental groups (Fig. 1A), i.e., FA-FA, O_3_-FA, FA-O_3_, and O_3_-O_3_, as described in the methods section. First, we monitored bodyweight change during 3-week recovery phase. As expected, the FA-exposed mice showed consistent weight gain (Fig. 1B). In contrast, mice exposed to 2ppm O_3_ exhibited a significant reduction in body weight gain within 2 days post-exposure, which slightly recovered by day 5, but was still significantly lower compared to FA-exposed mice at both day 5 and day 9 post-exposure (Fig. 1B). At day 21 post-exposure, the acute O_3_-exposed mice were able to recover from initial body weight losses (Fig. 1B).

To determine if the history of acute O_3_ exposure had any impact on the body weight change caused by subsequent repetitive O_3_ exposure, we also monitored body weight gain during the course of repetitive O_3_ exposures. The FA-FA group, i.e., mice without prior acute O_3_ exposure showed consistent body weight gain during the repetitive FA exposures, as expected (Fig. 1C). The O_3_-FA group, i.e., mice that had prior acute O_3_ exposure, showed slightly reduced body weight gain during the repetitive FA exposures, but the differences were not statistically significant (Fig. 1C). Both the FA-O_3_ and O_3_-O_3_ groups showed reduced body weight gains, following 2 repeated doses of O_3_. Interestingly, however, the reduction in the body weight gain was significantly less drastic in the O_3_-O_3_ group compared to the FA-O_3_ group. While the FA-O_3_ mice showed substantial recovery from body weight loss, mice in the O_3_-O_3_ group exhibited a comparatively modest increase in body weights (Fig. 1C). These data indicate that prior exposure to acute O_3_ impacts the change in body weight seen during repetitive O_3_- exposures.

### Prior exposure to acute O_3_ modulated BAL immune cell recruitment in repetitively O_3_-exposed mice.

To determine the effects of prior acute O_3_ exposure on lung’s immune responses to repetitive O_3_ exposure, bronchoalveolar lavage fluid (BALF) was assessed for the presence of immune cell composition and inflammatory mediators in lung airspaces. The airspace immune cell composition and counts were comparable between FA-FA and O_3_-FA groups. Both the groups contained macrophages as a predominant cell type in the BALF with minimal presence of granulocytes, i.e., neutrophils and eosinophils (Fig. 2A–E). As previously reported (16), mice in the FA-O_3_ group, which were exposed repetitively to O_3_ alone, exhibited increased immune cell counts (Fig. 2A–F), which was attributable to the increased counts of macrophages, neutrophils, eosinophils, and lymphocytes. Compared with the FA-O_3_ group, the total immune cell counts were reduced in O_3_-O_3_ group, although the difference did not reach statistical significance (Fig. 2B). However, as compared with the FA-O_3_ group, mice in the O_3_-O_3_ group exhibited significantly lower numbers of eosinophil (Fig. 2E; G) and lymphocyte populations (Fig. 2F), while neutrophil counts showed an upward trend (Fig. 2D). These data suggest that the three-week rest to acutely O_3_-exposed mice was sufficient to allow near complete restoration of normal immune cell composition, and that the prior acute O_3_ exposure modulates classical eosinophilic response of mice to repetitive O_3_ exposures.

Previously, we have shown that found in inflammatory zone 1 (FIZZ1) protein expression is elevated in the airway epithelium in repetitively O_3_-exposed mice (21, 22), suggesting its suitability as a marker of epithelial inflammation in response to repetitive O_3_ exposures. Consistent with this report, we found robust immunostaining for FIZZ1 in the airway epithelium of FA-O_3_ mice (Fig. 3A–C). However, the expression of FIZZ1 was reduced in mice from O_3_-O_3_ group, although the difference did not reach statistical significance (Fig. 3A–C). These data suggest suppressed inflammatory response localized to the airway epithelium.

### Prior exposure to acute O_3_ alters the levels of inflammatory mediators in airspaces of repetitively O_3_-exposed mice.

To assess the effect of prior acute O_3_ exposure on the release of inflammatory mediators into the airspaces of mice subjected to repetitive O_3_ exposure, we measured the levels of inflammatory mediators in the BALF harvested from FA-O_3_ and O_3_-O_3_ mice. As compared with the FA-O_3_ mice, the O_3_-O_3_ mice exhibited altered levels of inflammatory mediators. Eotaxin, IL-1α, and IL-1β levels were significantly reduced in the BALF from O_3_-O_3_ mice compared with FA-O_3_ mice (Fig. 4A–C). IL-12 p40 showed a downward trend in the BALF from O_3_-O_3_ mice compared with FA-O_3_ mice. (Fig. 4F). In contrast, G-CSF, KC, and IL-6 levels were significantly elevated in the BALF from O_3_-O_3_ mice compared with FA-O_3_ mice (Fig. 4D, E, G). Additionally, IL-10 exhibited an upward trend in O_3_-O_3_ mice compared with FA-O_3_ mice (Fig. 4E, H). These data indicate that prior acute O_3_ exposure alters the release of inflammatory mediators into the airspaces of mice subjected to repetitive O_3_ exposure.

### Prior exposure to acute O_3_ ameliorates mucous cell metaplasia in repetitively O_3_-exposed mice.

**Figure 5.**
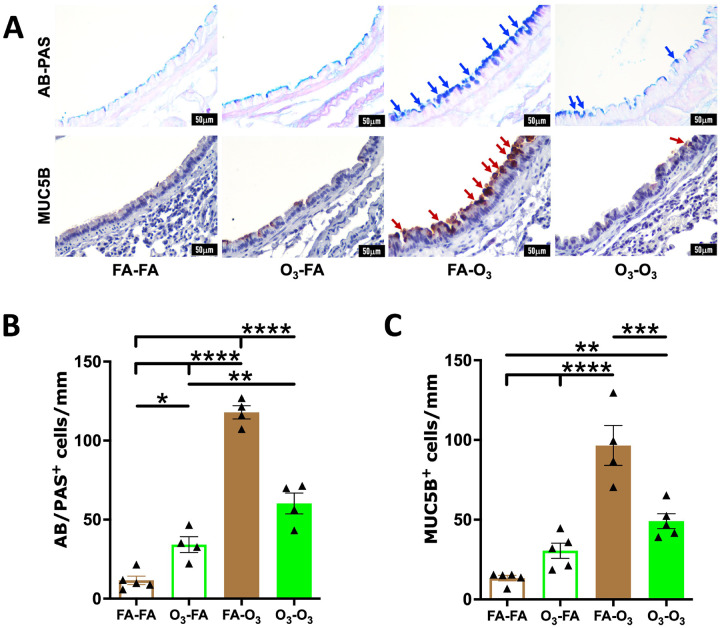
**Prior acute O_3_ exposure ameliorates mucous cell metaplasia in repetitively O_3_-exposed mice:**Representative photomicrographs from left lung lobe sections showing AB-PAS- (**A**; first row) and MUC5B- (**A**; second row) stained epithelial cells from mice in FA-FA, O_3_-FA, FA-O_3_, and O_3_-O_3_ experimental groups. Quantification of mucous cells **(B)** and MUC5B-positive cells **(C)** per millimeter of basement membrane from mice in FA-FA (open brown bar, *n* = 5), O_3_-FA (open green bar, *n* = 4–5), FA-O_3_ (solid brown bar, *n* = 4), and O_3_-O_3_ (solid green bar, *n* = 4–5) experimental groups. Error bars represent SEM. *p<0.05; **p<0.01; ***p<0.001; ****p<0.0001; using one-way ANOVA followed by Tukey’s multiple comparison post hoc test. Scale: 50mm. Magnification: 400X.

Previous reports suggest that repetitive O_3_ exposure results in type 2 (Th2) -like inflammatory responses, which is characterized by eosinophilic recruitment and mucous cell metaplasia (MCM) (4, 16, 22–24). Therefore, we assessed the effect of prior acute O_3_ exposure on MCM, a hall mark feature of repetitive O_3_ exposure (16, 22). We performed alcian blue-periodic acid Schiff (AB-PAS) staining and immunohistochemical localization of mucin protein MUC5B in lung sections of the four experimental groups of mice. As expected, the airway epithelial staining for AB-PAS and MUC5B in the FA-FA group confirmed the absence of MCM (Fig. 5A–C). Furthermore, the extent of AB-PAS and MUC5B staining were comparable between the FA-FA and the O_3_-FA groups (Fig. 5A–C). As previously reported (23), FA-O_3_ mice exhibited increased MCM, as indicated by significant increase in AB-PAS and MUC5B staining. However, the O_3_-O_3_ mice exhibited significantly reduced MCM, as indicated by diminished AB-PAS and MUC5B staining (Fig. 5A–C). These data suggest the suppression of MCM responses in mice that were previously subjected to acute O_3_-induced lung injury.

### Prior exposure to acute O_3_ results in lower mast cell counts and IL-4 production in repetitively O_3_-exposed mice.

**Figure 6.**
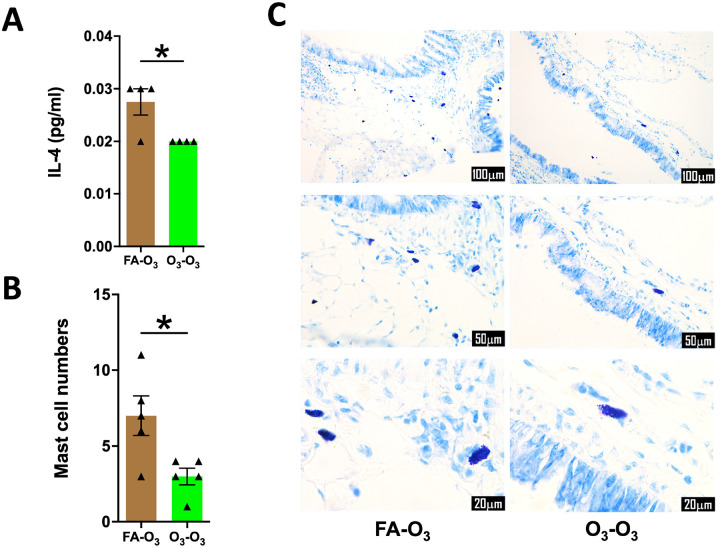
**Prior acute O_3_ exposure results in lower mast cell counts and IL-4 production in repetitively O_3_-exposed mice: (A)** IL-4 levels in mice in the BALF in FA-O_3_ (solid brown bar, *n* = 4) and O_3_-O_3_ (solid green bar, *n* = 4) experimental groups. **(B)** Quantification of mast cells in left lung lobe sections from mice in FA-O_3_ (solid brown bar, *n* = 5) and O_3_-O_3_ (solid green bar, *n* = 5) experimental groups. **(C)** Representative photomicrographs showing mast cells (violet color) in left lung lobe sections from mice in FA-O_3_, and O_3_-O_3_ experimental groups. Error bars represent SEM. *p<0.05; using Student’s t test. Scale: 100mm (first row), 50mm (second row), and 20mm (third row). Magnification: 200X (first row), 400X (second row) and 1000X (third row). IL-4, interleukin-4.

Based on our data highlighting altered Th2 response, i.e., reduced eosinophilia and diminished MCM in O_3_-O_3_ mice, we speculated that Th2 cytokine production would be diminished in the O_3_-O_3_ versus FA-O_3_ mice. First, we assessed the levels of IL-4, a Th2 cytokine and a ligand for IL-4Ra (25), in the BALF of O_3_-O_3_ mice versus FA-O_3_ mice. As anticipated, the levels of IL-4 were significantly reduced in the BALF from O_3_-O_3_ versus FA-O_3_ mice (Fig. 6A). Because IL-4 is expressed at a high level in the mast cells (26–28), we performed mast cell-specific special staining on the lungs of O_3_-O_3_ and FA-O_3_ mice. Consistent with the lower IL-4 levels, the mast cells were significantly less in O_3_-O_3_ versus FA-O_3_ mice (Fig. 6B–C).

Based on our findings showing the reduced levels of IL-4 production in O_3_-O_3_ mice compared with FA-O_3_ mice, we speculated that O_3_-O_3_ mice would have relatively lower number of IL-4Rα-expressing immune cells.

**Figure 7.**
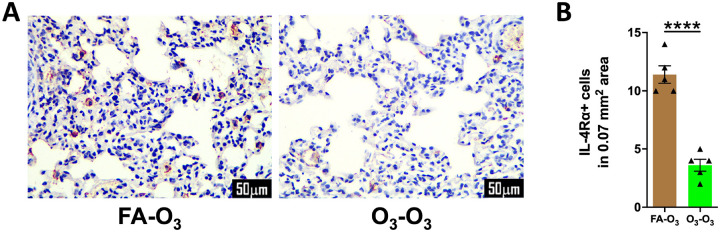
**Prior acute O_3_ exposure results in lower number of IL-4Ra+ immune cells in repetitively O_3_-exposed mice: (A)** Representative photomicrographs showing the immunolocalization of IL-4Ra in left lung lobe sections from mice in FA-O_3_ and O_3_-O_3_ experimental groups. **(B)** Quantification of IL-4Ra-stained cells per unit area analyzed in left lung lobe sections from mice in FA-O_3_ (solid brown bar, *n* = 5) and O_3_-O_3_ (solid green bar, *n* = 5) experimental groups. Error bars represent SEM. ****p<0.0001; using Student’s t test. Scale: 50mm. Magnification: 400X. IL-4Ra, interleukin-4 receptor alpha.

Lung cells expressing IL-4Rα are critical for the response to elevated levels of IL4 (25),. Therefore, we performed immunohistochemical localization of IL-4Rα in the left lung lobe sections of FA-O_3_ and O_3_-O_3_ mice. We observed significantly lower number of IL-4Rα-expressing immune cells in O_3_-O_3_ versus FA-O_3_ mice (Fig. 7A, B). These data suggest that the diminished Th2 response in O_3_-O_3_ mice is likely a result of diminished mast-cell recruitment, and, in turn, reduced production of IL-4 and reduced number of IL-4Rα-expressing immune cells.

## Discussion

Both acute high-concentration and repetitive low-concentration O_3_ exposures disrupt lung homeostasis and elicit contrasting inflammatory responses (10, 15, 16, 22). While acute high-concentration O_3_ exposure is characterized by neutrophilic inflammation that culminates in epithelial cell injury and desquamation (12, 29–34), the repetitive low-concentration O_3_ exposure induces Th2 inflammation followed by airway remodeling and is characterized by eosinophilic inflammation and MCM (16). The pulmonary consequences of both acute and repetitive O_3_ exposures have been studied extensively in humans and animal models (10, 12, 15, 16, 22, 35). Prior exposure to repetitive ozone has been shown to dampen acute ozone-induced lung inflammation (19). However, the impact of previous O_3_-induced acute lung injury on the subsequent development of repetitive O_3_-induced mucoinflammatory phenotype has not been directly investigated. To address this knowledge gap, we subjected C57BL/6J female mice to a single acute O_3_ exposure (2ppm for 3h), followed by a three-week recovery interval, and a subsequent repetitive O_3_ exposure (nine days; 1ppm for 4 hours per day). To our knowledge, this study is the first to evaluate the effects of previous O_3_-induced acute lung injury on the trajectory of mucoinflammatory responses to subsequent repetitive O_3_ exposure.

This study revealed several interesting findings: 1) mice with prior acute O_3_ exposure showed significantly reduced eosinophil and lymphocyte counts in the BALF following repetitive O_3_ exposure, indicating an attenuated recruitment of these immune cell types in lungs, 2) mice with prior acute O_3_ exposure had significantly lower numbers of AB-PAS-positive and MUC5B-positive airway epithelial cells following repetitive O_3_ exposure, indicating diminished MCM and MUC5B expression, 3) prior acute O_3_ exposure led to altered levels of multiple inflammatory mediators following repetitive O_3_ exposure, reflecting a reprogramming of airway inflammatory cytokine milieu, 4) prior acute O_3_ exposure inhibited repetitive O_3_-induced increase in epithelial expression of FIZZ1, a molecule linked to airway eosinophilia (36) and airway remodeling (37), and 5) prior acute O_3_ exposure mitigates the expression of IL-4 and IL4Ra suggesting suppressed activation of Th2 signaling pathway following repetitive O_3_ exposure. Collectively, comparison of repetitive O_3_-exposed mice with and without previous acute O_3_ exposure indicates that an initial acute O_3_ exposure can elicit long-term, possibly irreversible, changes that modify lung’s subsequent response to repetitive O_3_ exposure.

Airway eosinophilia is a characteristic pulmonary response to repetitive O_3_ exposure (4, 16, 38). Consistent with previous reports (16, 24, 38–40), FA-O_3_ mice in this study exhibited increased eosinophil counts. In contrast, the O_3_-O_3_ mice exhibited marked attenuation of eosinophilic inflammation, which was accompanied by decreased eotaxin levels. Eosinophil recruitment to the lung is largely driven by eotaxin, a robust eosinophil chemoattractant (41–43). Both alveolar macrophages and airway epithelial cells have been identified as important sources of eotaxin during O_3_-induced lung inflammation, suggesting that these cell types may contribute to the differences observed between FA-O_3_ and O_3_-O_3_ mice (43). How the prior acute O_3_ exposures suppress eotaxin expression programs in these cell types remains to be further investigated.

Acute pulmonary insults including O_3_ exposure (44, 45), and other stimuli such as LPS (18) and *Mycobacterium pneumoniae* infection (46) can cause an initial decline in resident alveolar macrophage numbers. Depletion of resident alveolar macrophage population is compensated by recruited bone marrow-derived monocytes that differentiate into alveolar macrophages (18, 47). This shift in alveolar macrophage ontogeny from fetal liver-derived to bone marrow-derived can alter macrophage functional programs and has been shown to modulate type-2 inflammatory responses, including attenuation of HDM-induced eosinophil recruitment (18). In the current study, total macrophage counts were comparable between FA-O_3_ and O_3_-O_3_ mice. However, O_3_-O_3_ mice had significantly fewer of IL-4Rα-expressing macrophages compared to FA-O_3_ mice. Myeloid IL-4Rα signaling is a critical regulator of eotaxin production and eosinophilic lung inflammation in mouse models (48, 49), providing a plausible mechanistic link between reduced IL-4Rα-expressing macrophages and diminished eosinophilia. Together, these findings suggest that prior acute O_3_ exposure may have changed the alveolar macrophage ontogeny and phenotype in O_3_-O_3_ mice, favoring bone marrow-derived macrophages with reduced IL-4Rα expression, and thereby abrogating repetitive O_3_ exposure-induced eosinophilia.

Airway epithelial cells are also a prominent source of eotaxin in the lung, therefore, a reduction in epithelial-derived eotaxin would be expected to reduce eosinophil recruitment (43). O_3_-O_3_ mice also exhibited lower levels of IL-1β and IL-4. IL-1β stimulates eotaxin production in airway epithelial cells (50), while IL-4 is a potent inducer of epithelial eotaxin and an important player in antigen- and OVA-induced eosinophilia (51–53). Therefore, reductions in these cytokines may have contributed to decreased epithelial eotaxin production. An alternative explanation is that prior acute O_3_ exposure induced long-term changes in epithelial signaling, reducing epithelial responsiveness to type-2 stimuli and thereby limiting eotaxin production. Future studies investigating the role of alveolar macrophages, airway epithelial cells, and cytokines implicated in eotaxin production, including IL-1β and IL-4, are warranted to further substantiate these mechanisms.

Repetitive O_3_ exposures induces MCM in the airway epithelium (16, 24, 38, 54–58). Consistent with these reports, we observed increased numbers of AB-PAS^+^ and MUC5B^+^ airway epithelial cells in FA-O_3_ mice. However, this response was attenuated in O_3_-O_3_ mice. The O_3_-O_3_ mice also displayed reduced lymphocyte numbers, lower IL-4 levels in the BALF, and reduced numbers of mast cells in the lungs, as compared to FA-O_3_ mice. Th2 cells and mast cells are well-established sources of IL-4 (26–28, 59). IL-4 is known to induce mucin gene expression and goblet cell-differentiation in the airway epithelial cells (60). Moreover, signaling through IL-4 receptor alpha (IL-4Rα) on airway epithelial cells is essential for allergen-induced mucus production, demonstrating that epithelial IL-4Rα signaling regulates MCM and mucin secretion (61). Additionally, IL-4 promotes the recruitment and differentiation of Th2 cells, which in turn drive mucus production via IL-4Rα-dependent pathways (62). Taken together, previous reports and our findings support a model in which prior acute O_3_ exposure suppresses repetitive O_3_-induced expansion of mast cells and/or Th2 cells, thereby reducing IL-4 levels and epithelial IL-4Rα signaling, which results in attenuated MCM.

In the current study, prior acute O_3_ exposure resulted in a relative increase in neutrophilic lung inflammation following repetitive O_3_ exposure. Multiple mechanisms likely contributed to the enhanced neutrophil infiltration. For instance, IL-6 and G-CSF have been shown to mediate neutrophil influx into the lungs (63, 64). Consistent with this, the O_3_-O_3_ group exhibited increased BALF levels of G-CSF and IL-6 as compared with the FA-O_3_ group. IL-4 induced IL-4Rα signaling can limit neutrophilic infiltration, whereas impairment of IL-4Rα signaling has been shown to exacerbate rhinovirus-induced neutrophil influx into the lung (65). Thus, increased IL-6 and G-CSF levels, accompanied by decreased IL-4 levels provide a plausible explanation for increased neutrophil infiltration in O_3_-O_3_ mice. Interestingly, a recent study demonstrated that prior repetitive low concentration O_3_ exposure prevents acute, high concentration O_3_-induced elevations in G-CSF and IL-6, corresponding with reduced neutrophilic recruitment (19). Taken together, previous studies and our findings indicate that the O_3_ concentration (high vs low) and duration (acute versus repetitive) used during the initial exposure modulates the epithelial response during subsequent exposures.

In summary, this study revealed several interesting findings. First, prior acute O_3_ exposure abolished the characteristic eosinophilic recruitment normally following repetitive O_3_ exposure, and this effect was accompanied by reduced levels of eotaxin in the airspaces. Second, prior acute O_3_ exposure exaggerated repetitive O_3_-induced neutrophilic inflammation. Third, prior acute O_3_ exposure diminished repetitive O_3_-induced MCM, which was accompanied by lower IL-4 levels, and decreased expansion of Mast cells and Th2 cells. Collectively, these novel findings indicate that O_3_-induced acute lung injury reprograms airway innate immune and epithelial responses to subsequent repetitive O_3_ exposures.
